# Supplementary material for: Sustained-input switches for transcription factors and microRNAs are central building blocks of eukaryotic gene circuits
Source: Genome Biol. 2013 Aug 23;14(8):R85. doi: 10.1186/gb-2013-14-8-r85 (PMC4054853; doi:10.1186/gb-2013-14-8-r85)
Supplement: Additional file 5 — HTML Browsable Motif Output. Zipped folder containing all WaRSwap and FANMOD motif output, viewable in a web browser. [file gb-2013-14-8-r85-S5.ZIP › HTML_browsable_motif_output/WaRSwap_ath_tair9/sigs_WSWRSLDraw_2-2000.pvals.heatmaps.html/motif_id_6_000000110_tftype_ath_upstream_-2000_0.html]

```
BG_MODEL = Rnew2
MOTIF_ID = 6_000000110
TF_TYPE = ath
UPSTREAM = -2000_0


PVals
FN_0.2	FN_0.4	FN_0.6	FN_0.8
dg_60.genes	0.831	0.96	0.952	0.001
dg_70.genes	0.833	0.954	0.952	0.001
dg_80.genes	0.826	0.958	0.953	0

ZScores
FN_0.2	FN_0.4	FN_0.6	FN_0.8
dg_60.genes	-1.033	-1.65	-1.629	2.394
dg_70.genes	-1.062	-1.632	-1.664	2.423
dg_80.genes	-1.011	-1.662	-1.678	2.482

StDevs
FN_0.2	FN_0.4	FN_0.6	FN_0.8
dg_60.genes	179.825	209.486	109.473	12.679
dg_70.genes	181.069	207.549	107.08	12.678
dg_80.genes	179.658	208.316	108.958	12.273
```
